# The Sweet Cherry Tree Genotype Restricts the Aggressiveness of the Wood Decay Fungi *Cytospora sorbicola* and *Calosphaeria pulchella*

**DOI:** 10.3390/microorganisms12122456

**Published:** 2024-11-29

**Authors:** Claudio Osorio-Navarro, Constanza Saez, Felipe Durán, Mauricio Rubilar, Paula Reyes-Bravo, Madelaine Azócar, Verónica Estrada, Marcela Esterio, Jaime Auger

**Affiliations:** 1 Departamento de Sanidad Vegetal, Facultad de Ciencias Agronómicas, Universidad de Chile, Santiago 8820808, Chile; anduin@ug.uchile.cl (C.O.-N.); constanza.saez@ug.uchile.cl (C.S.); felipe.duran.s@uchile.cl (F.D.); mrubilar@gmail.com (M.R.); paula.reyes.b@uchile.cl (P.R.-B.); madelaine.azocar@uchile.cl (M.A.); veronica.estrada@uchile.cl (V.E.); 2 Centre of Molecular Biology in Plants, Departamento de Biología, Facultad de Ciencias, Universidad de Chile, Santiago 7800003, Chile

**Keywords:** cherry tree varieties, cherry tree canker, vascular necrosis, PCR-HRM fungal detection

## Abstract

The wood decay fungi *Cytospora sorbicola* and *Calosphaeria pulchella* severely threaten the worldwide cultivation of sweet cherry trees (*Prunus avium* L.). Both fungi cause similar symptoms, including vascular necrosis, which leads to branch and twig dieback. In advanced stages of the disease, cankers are visible on tree branches and trunks. The sweet cherry is the most widely planted fruit tree in Chile, with 74,000 hectares in 2023. According to the planted surface, the predominant sweet cherry varieties are Lapins, Santina, Regina, and Bing. Variety-dependent susceptibility studies on *Cyt. sorbicola* and *Cal. pulchella* are lacking. The main entry points for wood necrosis-causing fungi are pruning wounds; therefore, we evaluated the aggressiveness of *Cyt. sorbicola* and *Cal. pulchella* in one-year-old sweet cherry plants. Santina and Lapins showed the lowest necrotic lesion caused by *Cyt. sorbicola* (13.6 and 14.31 mm, respectively), followed by Bing (19.51 mm) and Regina (26.14 mm). All plants infected by *Cyt. sorbicola* showed shoot blight regardless of the variety. In addition, there was a varying susceptibility to *Cal. pulchella*, with Lapins (21.6 mm), Bing (22.83 mm), Santina (27.62 mm), and Regina (30.8 mm) showing increasing levels of observed necrosis. The lesion caused by *Cal. pulchella* was more significant than that observed for *Cyt. sorbicola*, regardless of the cherry tree genotype. We identified each fungal growth from the wood necrosis progression area using two independent novel PCR-HRM strategies based on the *ITS* fungal region, which allowed us to differentiate each pathogen of interest individually or simultaneously. This study demonstrates different levels of susceptibility of sweet cherry tree genotypes to wood-degrading pathogens, emphasizing the need to include these factors in phytosanitary management programs.

## 1. Introduction

The fungal canker pathogens *Cytospora sorbicola* (*Cyt. sorbicola*) and *Calosphaeria pulchella* (*Cal. pulchella*) pose a significant phytosanitary threat to fruit trees orchards worldwide, including sweet cherry trees (*Prunus avium* L.). Both pathogens cause wood necrosis in sweet cherry trees, resulting in the decay of branches, twigs, and trunks, impacting orchard longevity and productivity [1,2,3,4]. To infect their hosts, *Cyt. sorbicola* and *Cal. pulchella* require a wound [5]. While pruning wounds have been considered the primary cause of fungal infection in sweet cherry trees, recent research indicates that natural and fruit harvesting wounds are also critical infection points [5]. *Cyt. sorbicola* and *Cal. pulchella* cause similar longitudinal necrosis from pruning wounds. However, different infection strategies have been proposed for these pathogens based on the symptoms observed in several woody plants. *Cytospora* species colonize the subcortical bark tissue and then spread toward the wood pith [2,6]. Necrosis by *Cal. pulchella* begins at the pith and gradually spreads to the xylem, cambium, phloem, and cortical tissues [7]. In trees where the infection has progressed severely, multiple *Cyt. sorbicola* pycnidia or *Cal. pulchella* perithecia can be observed beneath the bark. Consequently, overwintering spores of both pathogens serve as the primary source of inoculum for initiating new infections.

Chile leads sweet cherry exports in the Southern Hemisphere, with 415,394 metric tons exported in the 2023 season [8]. Sweet cherry orchards cover 74,000 hectares, making it the country’s leading fresh fruit crop [9]. Four sweet cherry tree varieties contributed 84% of the total production in 2023: Lapins (36%), Santina (27%), Regina (15%), and Bing (6%) [9]. The cultivation area is concentrated in the Chilean Central Valley, which has a Mediterranean climate, ensuring the crop’s temperature and water requirements are met. These varieties produce large fruits in colors ranging from dark red to deep purple, with Bing cherries serving as a standard for color and flavor even a century and a half after the variety was bred. The genetics of the sweet cherry tree varieties determine some agronomically relevant characteristics. Lapins and Santina are self-fertile varieties, while Regina and Bing require cross-pollination. Furthermore, in this group, Santina is an early-season variety, followed by Lapins and Bing as a mid-season variety and Regina as a late-season variety. In addition, Lapins are known for having the best compatibility with rootstocks [10,11]. However, differences in pathogen responses have not been thoroughly researched. 

The area planted with sweet cherry trees increased 19 times in 20 years in Chile. High-density orchards have accompanied the expansion of this crop. However, only recently have *Cytospora* and *Calosphaeria* canker been formally reported as a distinct issue in sweet cherry trees from Chilean orchards [1,4]. Symptoms such as shoot dieback, wood decay, and gummosis have confused previous diagnoses due to their similarity with bacterial canker caused by *Pseudomonas syringae* pv. *syringae* [12]. *Cyt. sorbicola* and *Cal. pulchella* can be found in young or mature orchards, even coexisting in the same cherry tree. Managing these fungal canker pathogens is primarily preventive in cherry tree orchards. Pruning wounds are treated with pruning pastes, with or without fungicides selected by in vitro tests. Removing pruning material is also crucial to reducing inoculum sources. In addition, applying biological control microorganisms such as *Trichoderma* spp. to restrict inoculum sources has shown promising results in *Prunus* spp. [13,14]. However, the reference *Trichoderma*, *Trichoderma atroviride*, has low efficacy against *Cal. pulchella* in the specific case of cherry trees [15]. The only available strategy for managing infected plants is to remove visibly necrotic tissues. In addition, biological differences between *Cyt. sorbicola* and *Cal. pulchella*, including optimal growth temperatures, spore production, and spore release, prevent a universal field management system [16]. Therefore, efficient field management of these canker-causing pathogens requires developing and implementing tools that facilitate their rapid and accurate differentiation. Specific polymerase chain reaction (PCR)-based methods have been developed to detect canker pathogens in *Prunus* spp., including *Cytospora* spp. [17,18]. However, they are inefficient as they require testing each fungal microorganism with species-specific PCR. In situ strategies based on loop-mediated isothermal amplification (LAMP) have been developed to detect *Cytospora* species [19]. Although their contribution is significant in determining the health of asymptomatic plants, the technique is limited to one single species. In contrast, the detection of *Cal. pulchella* is still pending. The high-resolution melting (HRM) analysis is a specific and highly sensitive post-PCR method based on the identity of a DNA fragment. The nucleotide content of the fragment determines the energy required to break the hydrogen bonds forming the double strand. By applying a temperature gradient with a DNA-intercalating fluorophore, specific denaturation profiles can be obtained, essentially functioning as the fragment fingerprint [20]. Accordingly, a single nucleotide difference between two DNA fragments can be distinguished by PCR-HRM. PCR-HRM is successfully employed to detect food, human, animal, and plant pathogens [21,22,23,24,25]. Remarkably, PCR-HRM is versatile for detecting multiple microorganisms or genotypes in a single reaction. Hypervariable DNA regions flanked by conserved regions enable the simultaneous identification of distant organisms using a single set of primers. Therefore, the costs related to diagnosing pathogens using PCR-HRM are considerably reduced.

This study aimed to assess the susceptibility of the most agriculturally significant sweet cherry tree varieties to *Cyt. sorbicola* and *Cal. pulchella*. Additionally, a novel PCR-HRM tool was designed and implemented to quickly and precisely detect these canker-causing fungi. The successful diagnosis of *Cyt. sorbicola* and *Cal. pulchella* and the subsequent implementation of pathogen/variety-specific management programs have the potential to significantly reduce disease incidence in the field, thereby enhancing the productivity and longevity of sweet cherry tree orchards.

## 2. Materials and Methods

### 2.1. Sweet Cherry Orchards Survey and Sampling

During the 2020–2021 growing season, symptoms associated with wood decay fungi in sweet cherry tree orchards of Santina, Lapins, Bing, and Regina varieties were screened. The orchards were located in the Chilean Central Valley, including the Metropolitan, O’Higgins, and Maule regions (*n* = 3 per variety). Symptomatic wood was collected from Lapins sweet cherry trees with signs of *Cytospora* sp. (presence of pycnidia). The wood bordering the necrosis area was cut into 5 mm^2^ pieces. Then, they were treated in 1.5% sodium hypochlorite for 1 min for surface disinfection and flamed for 15 s. The wood pieces were plated on 60 mm Petri dishes containing 0.5 g/L streptomycin-amended potato dextrose agar (PDA) medium. After one week, the growing mycelium from the wood pieces was recovered and plated on a fresh antibiotic-amended PDA medium. Colonies were allowed to grow until sporulation (10 to 15 days). Then, single-conidia cultures were generated.

### 2.2. Molecular Identification of Cyt. sorbicola

Three-day-old isolates of *Cytospora* sp. generated from a single conidia were used for species identification. Genomic DNA was isolated using the DNeasy Plant Mini Kit following the manufacturer’s instructions (QIAGEN, Hilden, Germany). The *Internal Transcribed Spacer* (*ITS*) region and gene fragments of *Actin* (*ACT*) and *Translation elongation factor 1-alpha* (*TEF-1α*) were amplified employing the primers *ITS1F*/*ITS4*, *ACT512F*/*ACT-783R*, and *EF1-728*/*EF1-986*, respectively [26,27]. The PCR mix was composed of 12 ng genomic DNA, 1X GoTaq^®^ Green Master Mix (Promega, Madison, WI, USA), and 100 nM of each primer; 20 μL volume was completed with nanopure water (Promega, Madison, WI, USA). The PCR conditions consisted of a 5 min denaturation step at 95 °C, followed by 40 cycles of 30 s at 95 °C, 30 s at 55 °C, and 30 s at 72 °C for *ITS*; 40 s at 95 °C, 35 s at 55 °C, and 1 min at 72 °C for *ACT*; and 40 s at 95 °C, 30 s at 52 °C, and 1 min at 72 °C for *TEF-1α*, with a final extension of 7 min at 72 °C. The PCR product was evaluated in an agarose gel (Lafken, Fermelo Biotec, Santiago, Chile), purified, and used for sequencing (Psomagen, Rockville, MD, USA). Sequences were analyzed in the BioEdit software version 7.2.5 [28] to obtain consensus sequences for each gene fragment. Then, each group of sequences was aligned through the ClustalW program integrated into the BioEdit software and refined independently under the G-Block algorithm, conditioning the less strict block option to establish the most informative regions for subsequent phylogenetic analysis [29]. The regions were concatenated manually. Then, phylogenetic analyses were conducted in MEGAX [30]. Specifically, the maximum likelihood method, the general time-reversible nucleotide substitution model (GTR model), gamma distribution, and a Bootstrap of 1000 repetitions were used. The analysis included sixteen species (Table 1), and the tree was rooted (out-group) to *Diaporthe vaccinii* (CBS 160.32) [31].

### 2.3. Growth and Cultivation Conditions of Cyt. sorbicola and Cal. pulchella

*Cyt. sorbicola* CY1 and *Cal. pulchella* CpL01 isolates were grown on a PDA medium at 20 °C in continuous darkness. Cultures were refreshed every 15 days by transferring 5 mm diameter sections from the active growth zone of the colony to plates with fresh PDA medium.

### 2.4. In Planta Infection Assays

For the *Cyt. sorbicola* infection assay, 20 sweet cherry trees of the Santina, Lapins, Bing, and Regina varieties were acclimatized for six months in the O’Higgins region (34°42′51.3″ S 71°15′11.2″ W). All plants used were one year old and 1.5 m tall. The trees were cut down to 80 cm and inoculated with the infection or control treatment. The plants were evaluated after six months. A volume of 10 µL at a concentration of 3 × 10^5^/mL conidia was inoculated into the wounds generated in the plant material. As a control, 10 µL of sterile distilled water was inoculated. For each variety, ten plants were used for the control treatment and ten for the pathogen infection. Vaseline was used to cover the wound after the treatments, preventing nonspecific infections and moisture loss. The wound was then sealed with parafilm.

For the *Cal. pulchella* infection assay, 20 cm long branches were obtained from independent plants, 20 for each variety of sweet cherry tree under study. A wound of 60 mm in diameter was produced under the bark, where the infection or control treatment was inoculated. The infection and control treatment were generated using the same methodology previously described for *Cyt. sorbicola*. The inoculated branches were kept in a humid chamber at 25 °C. The assay was evaluated after one month.

### 2.5. Design and Validation of the Cyt. sorbicola and Cal. pulchella Identification System by PCR-HRM

The *ITS* region of *Cyt. sorbicola* isolates obtained in this study and those of *Cal. pulchella* previously obtained by our group were aligned in the BioEdit software [28]. The *ITS* regions of the isolates described in the first report of *Cyt. sorbicola* in sweet cherry trees and *Cal. pulchella* in peach trees from Chile were included [4,32]. Conserved DNA segments between the *ITS* sequences of both species were examined manually. Primers were designed based on the *ITS*-conservated regions (Table 1), and two different PCR-HRM strategies were conceived. First, a universal primer system (*Calo-Cytos For*(*ITS*) and *Calos-Cytos Rev*(*ITS*) primers) was used to detect *Cyt. sorbicola* and *Cal. pulchella* simultaneously. Alternatively, a system using a universal forward primer (*Calo-Cytos_For-univ1*(*ITS*)) with pathogen-specific reverse primers (*Cyto_Rev-esp*(*ITS*) and *Calo_Rev-esp*(*ITS*)) was used. In the last strategy, the primer pairs produced DNA fragments of different sizes: 114 and 85 bp for *Cyt. sorbicola* and *Cal. pulchella*, respectively. The primer sequences used are detailed in Table 2. The annealing temperature for the designed primers was determined using temperature-gradient PCR in a T-100 model thermocycler (BioRad, Hercules, CA, USA). Then, the sensitivity and efficiency of the primers were determined using a DNA concentration gradient, where the primers reached an efficiency of 99%, defined through the LinRegPCR software version 2021.2 [33]. The PCR mix was composed of 12 ng genomic DNA, 1X SensiFast Master Mix containing the third-generation fluorescent dye EvaGreen (Bioline, Meridian Bioscience, Cincinnati, OH, USA), and 100 nM of each primer; the volume was completed with nanopure water (Promega, Madison, WI, USA). The PCR protocol was performed in a 72-well carousel in a Rotor-Gene Q 2plex (QIAGEN) PCR-HRM thermocycler (QIAGEN, Hilden, Germany). A fast PCR protocol was used with the following parameters: (i) For the universal primers system, an initial denaturing step of 95 °C for 5 min was used, followed by 25 cycles at 95 °C for 30 s and 64 °C for 30 s. HRM was performed from 80.5 to 85.5 °C, rising by 0.05 °C in each step. (ii) For pathogen-specific reverse primers, an initial denaturing step of 95 °C for 5 min was used, followed by 30 cycles at 95 °C for 30 s and 62 °C for 30 s. HRM was performed from 76 to 80 °C, rising by 0.05 °C in each step. Both protocols considered the default parameters of 2 s hold time for each step and 90 s pre-melt conditioning on the first step. The Rotor-Gene Q Series software version 2.3.5 was used for data analysis.

### 2.6. Recovery of Cyt. sorbicola and Cal. pulchella from Wood Pieces

Wood fragments of 5 mm^2^ were collected from the area adjacent to the necrosis developed in plants infected with *Cyt. sorbicola* and *Cal. pulchella*. Wood pieces were also obtained from plants under control treatment. For each variety, 60 pieces were generated in total. Pieces were disinfected in a 1.5% sodium hypochlorite solution for 1 min, flamed for 15 s, and plated on 60 mm Petri dishes containing 0.5 g/L streptomycin-amended PDA medium. After four days, fungal mycelium growing out of the wood pieces were collected. Then, the DNA was isolated, and the PCR-HRM pathogen-specific reverse primer system was utilized.

### 2.7. Statistical Analysis

Statistical analyses were conducted using ANOVA and the Tukey post hoc test in the InfoStat software 2017 version [34]. Differences between PCR-HRM profiles were determined using the Rotor-Gene Q Series software version 2.3.5 (QIAGEN, Hilden, Germany) with a closed threshold at 98% confidence. Data were plotted in GraphPad Prism v.9.4.0 (San Diego, CA, USA).

## 3. Results

### 3.1. Cyt. sorbicola and Cal. pulchella Are Widespread in Sweet Cherry Orchards from the Chilean Central Valley

**Figure 1:**
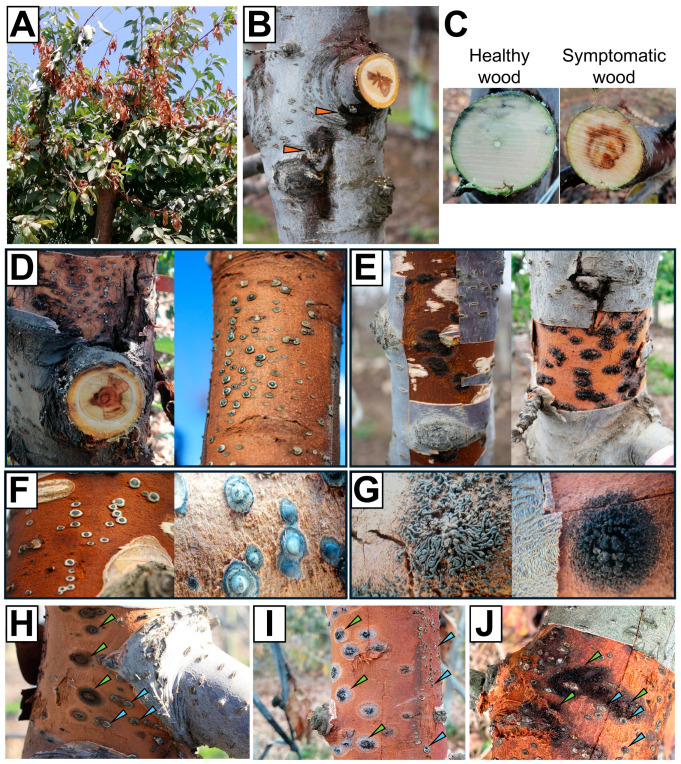
Symptoms and signs of *Cytospora* sp. and *Calosphaeria* sp. in sweet cherry trees. (**A**). Branch and twig dieback in adult trees during the productive stage. (**B**). Wood necrosis accompanied by exudations (orange arrowheads). (**C**). Cross section of healthy and symptomatic branches showing changes in tissue coloration. (**D**,**F**). *Cytospora* sp. signs in adult trees. (**E**,**G**). *Calosphaeria* sp. signs in adult trees. (**H**–**J**). *Cytospora* sp. (light blue arrowheads) and *Calosphaeria* sp. (green arrowheads) can be observed coexisting on the same host.

**Figure 2:**
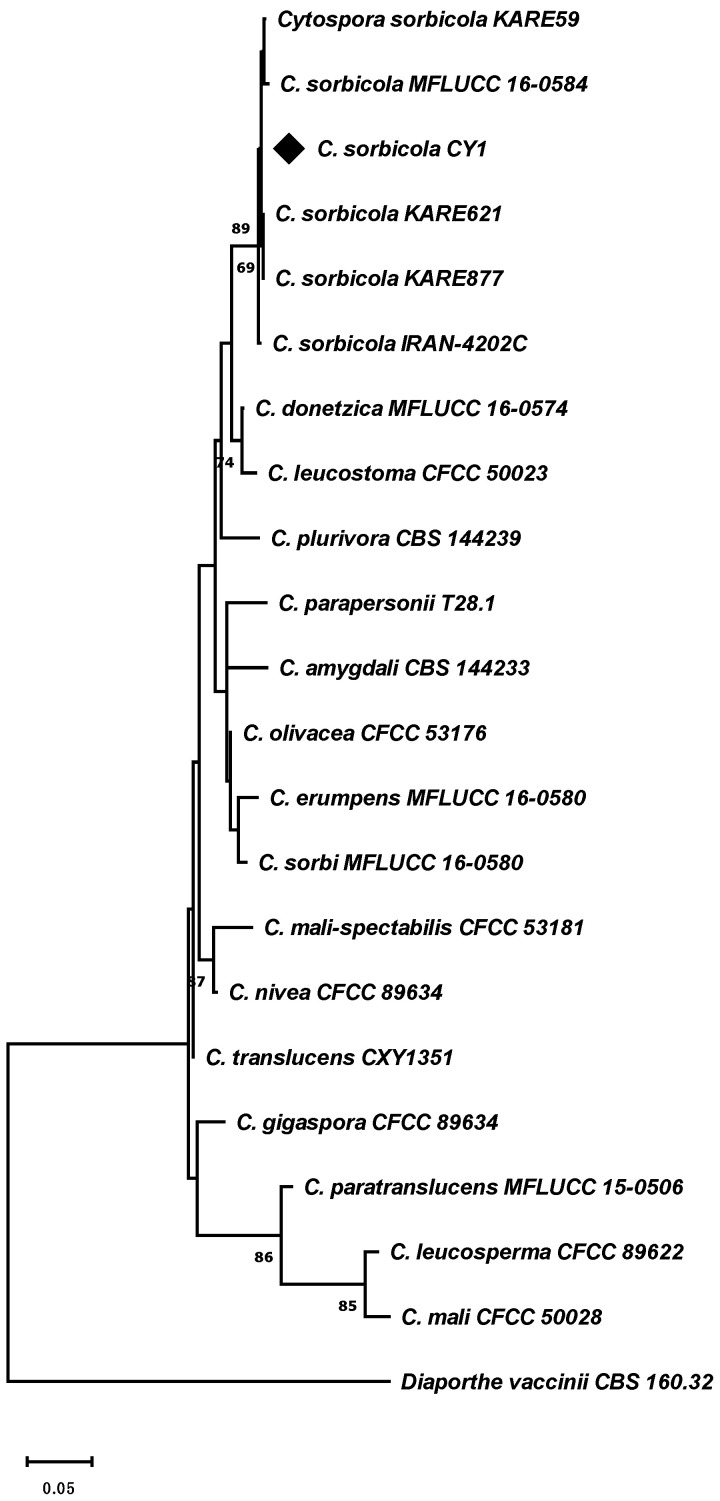
*Cytospora sorbicola* identification by molecular phylogeny. A phylogenetic tree was generated from a maximum-parsimony analysis of three Chilean isolates and 16 *Cytospora* species. The tree was inferred from a three-locus data set (*ITS*-*ACT*-*TEF-1α*). The numbers above branches represent bootstrap values from 1000 replicates. The diamond indicates a *Cyt. sorbicola* representative Chilean isolate. *Diaporthe vaccinii* strain CBS 160.32 was used as an outgroup.

The sweet cherry orchards (*n* = 3 per variety) surveyed in the Central Valley of Chile exhibited at least one tree with symptoms associated with wood decay fungi with signs of *Cytospora* sp. or *Calosphaeria* sp. Wood necrosis, along with exudates or gummosis in some cases, was noticed during the growing season in branch dieback (Figure 1A,B). The wood showed browning and necrosis in the pith, affecting different tissues to varying degrees. Healthy wood, on the other hand, did not exhibit oxidation or browning (Figure 1C). *Cytospora* sp. pycnidia (Figure 1D,F) or *Calosphaeria* sp. perithecia (Figure 1E,G) could be observed beneath the bark in symptomatic trees. Notably, *Cytospora* sp. and *Calosphaeria* sp. could coexist within the same plant. Pycnidia and perithecia were observed occupying neighboring domains of the plant trunk (Figure 1H–J). *Cytospora* sp. isolated from symptomatic wood was identified as *Cyt. sorbicola* by multilocus molecular phylogeny (MMF) (*n* = 3) with a three-locus data set (*ITS*-*ACT*-*TEF-1α*) (Figure 2). For this study, we utilized the *Cyt. sorbicola* isolate called CY1, identified by MMF, and the *Cal. pulchella* CpL01 isolate, previously characterized by Auger et al. (2020) [1], from the same geographic location.

### 3.2. Regina Is the Sweet Cherry Variety Most Susceptible to Cyt. sorbicola and Cal. pulchella

**Figure 3:**
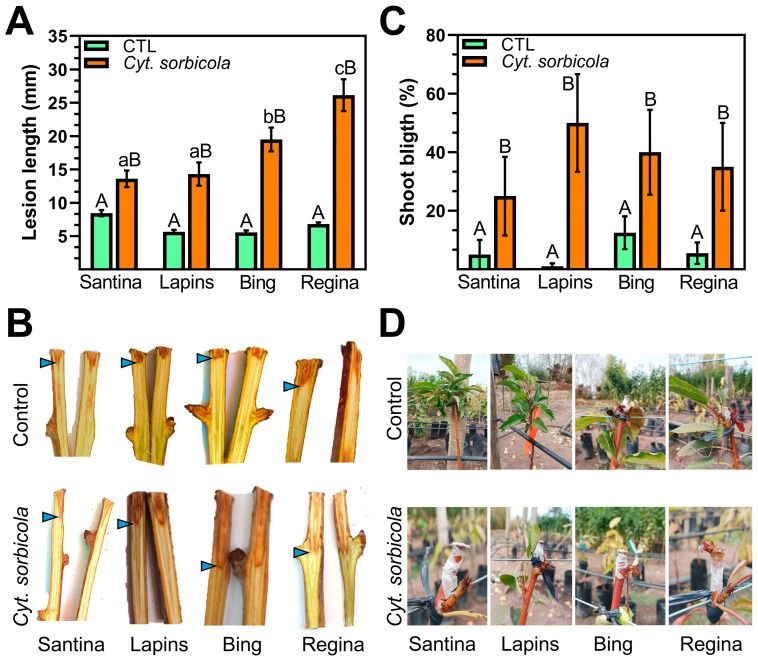
Susceptibility of sweet cherry varieties Santina, Lapins, Bing, and Regina to *Cyt. sorbicola*. (**A**,**B**). Lesions in one-year-old trees were evaluated after six months of control treatment (water) or infection treatment (*Cyt. sorbicola* conidia suspension). (**C**,**D**). The shoot blight resulting from infection treatment was measured. The lesion limit is highlighted by the blue arrowheads in (**B**). Error bars represent standard error. Different letters represent significant differences according to Tukey’s HSD test (*p* > 0.05). Upper- and lower-case letters represent independent statistical analyses.

One-year-old sweet cherry trees inoculated with *Cyt. sorbicola* developed wood necrosis. The pathogen-induced necrosis was significantly higher than the control condition in each sweet cherry variety evaluated (Figure 3); however, the response between sweet cherry genotypes was heterogeneous. The lesion length generated by *Cyt. sorbicola* was significantly greater in Regina and Bing, with average lesion sizes of 26.14 and 19.51 mm, respectively (Figure 3). The observed necrosis in Santina and Lapins averaged 13.6 and 14.1 mm, respectively, significantly lower than the remaining evaluated varieties but 39% and 61% greater than the control treatment. The evaluated sweet cherry varieties responded similarly to mechanical damage stress, as shown by the lesion length under control treatment.

Plant top remotion leads to the loss of apical dominance, prompting lateral sprouting. Shoot and twig blight is a common symptom of plants infected with wood decay fungi. Hence, the death of new shoots was assessed following the *Cyt. sorbicola* infection. Shoot blight was significantly higher in all infected plants compared to the control treatment, regardless of the sweet cherry variety (Figure 3). Lateral buds developed smaller leaves that brown and split early throughout the whole plant. Notably, the shoot blight percentage was statistically equivalent among the sweet cherry varieties in the study, suggesting a conserved program of lateral organ development in response to infection (Figure 3).

**Figure 4:**
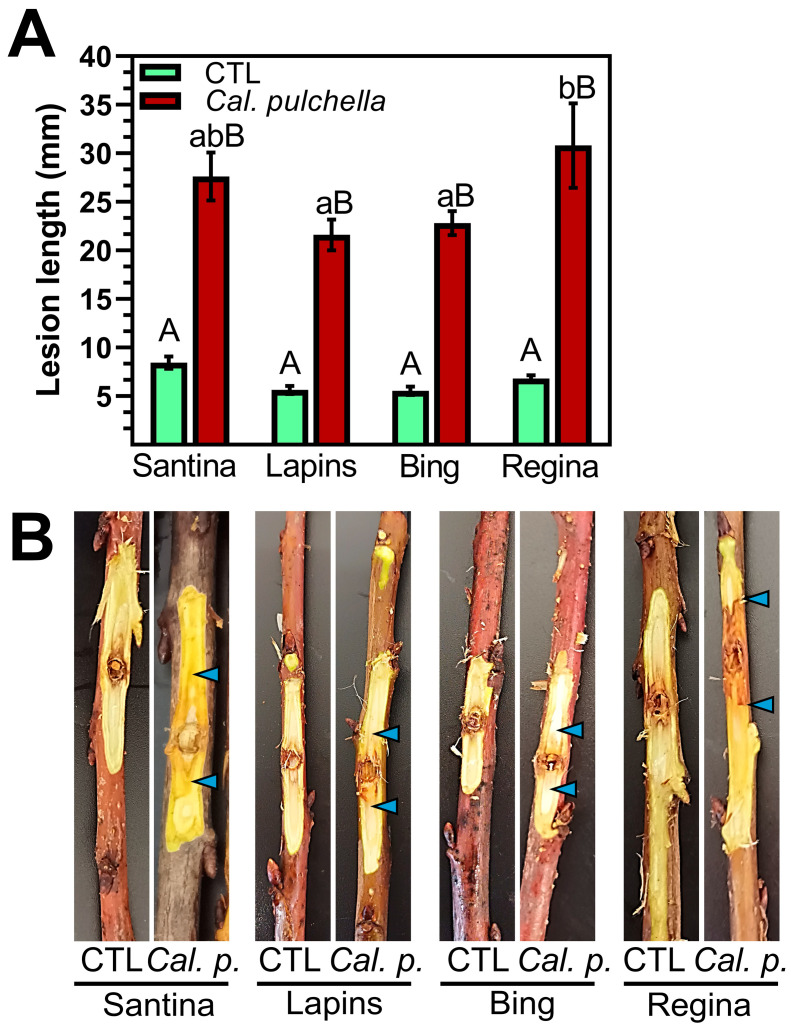
Susceptibility of sweet cherry varieties Santina, Lapins, Bing, and Regina to *Cal. pulchella*. (**A**,**B**). Lesions in branches from one-year-old trees were evaluated after one month of control treatment (water) or infection treatment (*Cal. pulchella* conidia suspension). The lesion is delimited by the blue arrowheads in (**B**). Error bars represent standard error. Different letters represent significant differences according to Tukey’s HSD test (*p* > 0.05). Upper- and lower-case letters represent independent statistical analyses.

Necrosis caused by *Cal. pulchella* was significantly more extensive than the control treatment. On average, the lesion caused by the pathogen was four times bigger than the control treatment (Figure 4), exceeding the 2.8-fold average observed by *Cyt. sorbicola* infection. Remarkably, the mechanical wound caused by the control treatment was the same for both experimental conditions: 6.612 vs. 6.613 mm on average. The sweet cherry varieties Lapins and Bing exhibited minor necrosis caused by *Cal. pulchella*, measuring 21.6 and 22.8 mm, respectively. Necrosis increased significantly in the Santina variety with 27.62 mm. However, the most severe necrosis recorded within the group of sweet cherry tree varieties studied was 30.8 mm on average in the Regina variety (Figure 4).

Therefore, the Regina variety was consistently the most susceptible to fungi that cause wood cankers. The Santina variety was an exceptional case, with a lower susceptibility to *Cyt. sorbicola* but a wound equivalent to that of the Regina variety by *Cal. pulchella*, suggesting a differential response mechanism for these pathogens. Meanwhile, the Lapins variety was less susceptible to *Cyt. sorbicola* and *Cal. pulchella.* The Bing variety, in contrast, showed an intermediately high susceptibility among the studied sweet cherry genotypes.

### 3.3. Implementation of a System for Cyt. sorbicola and Cal. pulchella Identification by PCR-HRM

**Figure 5:**
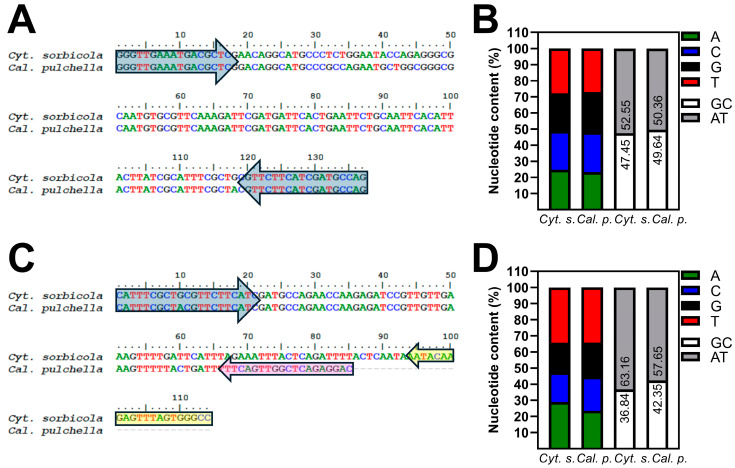
Primer design for PCR-HRM assays. (**A**). The universal detection system employed primers *Calo-Cytos For*(*ITS*) and *Calos-Cytos Rev*(*ITS*) to produce a 137 bp fragment in *Cyt. sorbicola* and *Cal. pulchella*. (**C**). The pathogen-specific reverse primer system employed the *Calo-Cytos_For-univ1*(*ITS*) (blue arrow), *Cyto_Rev-esp*(*ITS*) (yellow arrow), and *Calo_Rev-esp*(*ITS*) (magenta arrow) primers to obtain fragments of 114 and 85 bp from *Cyt. sorbicola* and *Cal. pulchella* DNA, respectively. (**B**,**D**). The fragments obtained vary in their nucleotide content.

The identification of wood-colonizing fungi presents several challenges. In situ detection depends on DNA isolation, restricted by the wood’s lignin and other phenolic compounds and the underrepresentation of fungal DNA within the sample. In vitro isolation requires long periods for the fungus to grow and develop spores and/or reproductive structures that allow its morphological recognition. Furthermore, finding multiple wood-living fungi within the same host is possible. Indeed, *Cyt. sorbicola* and *Cal. pulchella* can coexist on the same sweet cherry tree (Figure 5). Hence, a PCR-HRM strategy was designed to identify *Cyt. sorbicola* and *Cal. pulchella*, reducing diagnosis time.

*Cyt. sorbicola* and *Cal. pulchella* are phylogenetically distant, belonging to Cytosporaceae (order Diaporthales) and Calosphaeriaceae (order Calosphaeriales), respectively. However, exploring the *Internal Transcribed Spacer* (*ITS*) genomic region from both pathogens allowed the design of two different PCR-HRM strategies based on conserved DNA regions (Figure 5). The *Calo-Cytos For*(*ITS*) and *Calos-Cytos Rev*(*ITS*) universal primers amplified a 137 bp fragment from the *ITS* region. The nucleotide content of the fragment was species-specific, with 47.45% of CG and 52.55% of AT for *Cyt. sorbicola* and 49.64% of CG and 50.36% of AT for *Cal. pulchella* (Figure 5A). The second strategy, which employed species-specific reverse primers, was developed to verify and strengthen the diagnosis results. The forward primer *Calo-Cytos_For-univ1*(*ITS*) with the *Cyt. sorbicola*-specific reverse primer *Cyto_Rev-esp*(*ITS*) generated a 114 bp fragment, while the *Cal. pulchella*-specific reverse primer *Calo_Rev-esp*(*ITS*) generated an 85 bp fragment from the *ITS* region. Each fragment differed in nucleotide content; the *Cyt. sorbicola* fragment contained 36.84% of CG and 63.16% of AT, and the *Cal. pulchella* fragment contained 42.35% of CG and 57.65% of AT (Figure 5B). Differences in nucleotide content are critical to the success of the PCR-HRM strategy.

**Figure 6:**
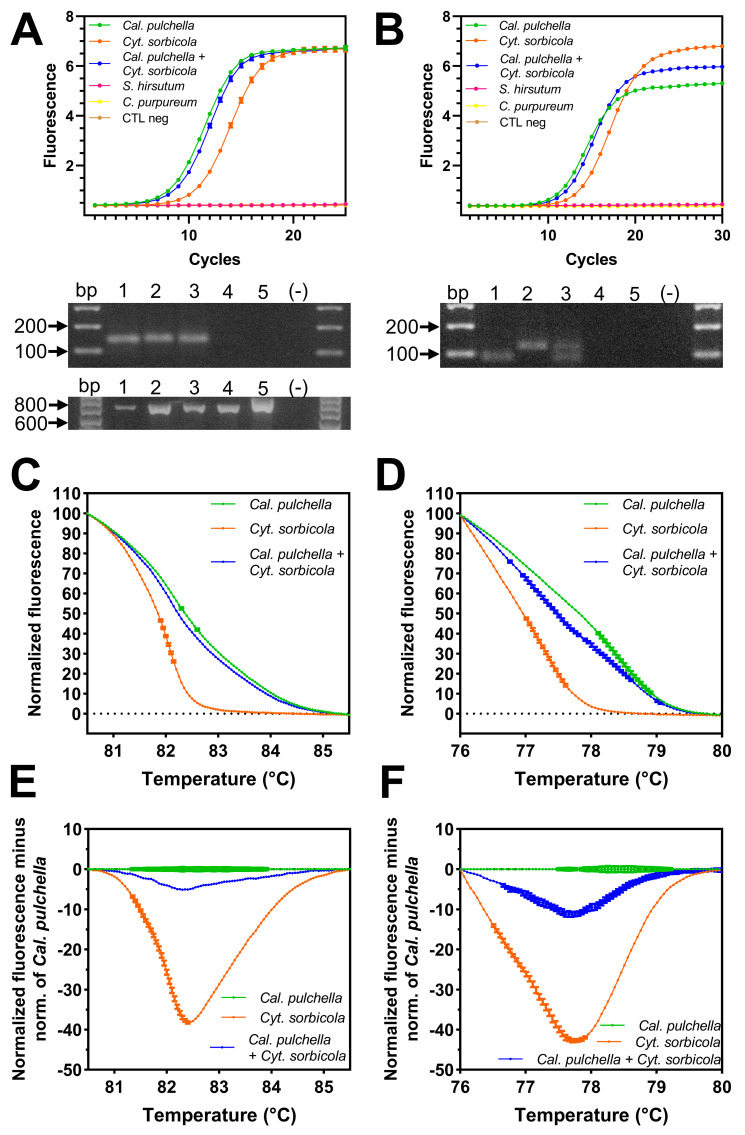
*Cyt. sorbicola* and *Cal. pulchella* detection by PCR-HRM. (**A**,**B**). Amplifications by the universal (**A**) or pathogen-specific reverse primer system (**B**) were specific to *Cyt. sorbicola* or *Cal. pulchella*. Neither system could use DNA from other common wood fungi, such as *Stereum hirsutum* (*S. hirsutum*) or *Chondrostereum purpureum* (*C. purpureum*). The universal system led to a 137 bp fragment being obtained (**A**). Amplification of the *ITS* region from all fungi used was amplified as a positive control (**A**). The pathogen-specific primer reverse system generated fragments of 114 and 85 pb for *Cyt. sorbicola* and *Cal. pulchella*, respectively (**B**). From agarose gels: 1 = *Cyt. sorbicola*, 2 = *Cal. pulchella*, 3 = *Cyt. sorbicola* + *Cal. Pulchella*, 4 = *S. hirsutum*, 5 = *C. purpureum*, (-) = negative control. (**C**,**D**). Normalized HRM profiles of *Cyt. sorbicola*, *Cal. pulchella*, and *Cyt. sorbicola* + *Cal. pulchella* by the universal (**C**) or pathogen-specific reverse primer system (**D**). (**E**,**F**). Difference curves of HRM using *Cal. pulchella* as the reference baseline curve by the universal (**E**) or pathogen-specific reverse primer system (**F**).

The primer pairs designed for each strategy (universal or reverse-specific primers) amplified the expected fragments from *Cyt. sorbicola* and *Cal. pulchella* DNA, even from the mixture of DNA of both species (*Cyt. Sorbicola* + *Cal. pulchella*) (Figure 6A,B). None of the designed systems was able to amplify a DNA fragment from the genomic DNA of cosmopolitan microorganisms frequently found in *Prunus* spp. wood, such as *Stereum hirsutum* and *Chondrostereum purpureum* (Figure 6A,B). The *ITS* region of each microorganism tested was amplified using the *ITS1F* and *ITS4* primers as a DNA amplification control (Figure 6A). Therefore, the amplification was specific to *Cyt. sorbicola* and *Cal. pulchella.*

The PCR-HRM analysis produced three distinct curves individualizing *Cyt. sorbicola*, *Cal. pulchella*, and *Cyt. sorbicola* + *Cal. pulchella* (Figure 6C,D). The differences between the profiles of *Cyt. sorbicola* and *Cyt. sorbicola* + *Cal. pulchella* were more evident under the reverse-specific primer strategy under the normalized fluorescence criteria (Figure 6D). However, the difference graph generated using the *Cal. pulchella* profile as the baseline in universal or reverse-specific primer strategies revealed that *Cyt. sorbicola* and *Cyt. sorbicola* + *Cal. pulchella* profiles deviated significantly (Figure 6E,F), indicating that the profiles were indeed different and facilitating the discrimination of *Cyt. sorbicola* and *Cal. pulchella*.

### 3.4. Cal. pulchella Colonizes Sweet Cherry Wood More Effectively than Cyt. sorbicola

Wood pieces (*n* = 60) were recovered from the area adjacent to the necrosis of each plant infected with *Cyt. sorbicola* or *Cal. pulchella* to evaluate the pathogen’s development. After four and seven days of incubation, fungal growth was recovered from the wood and analyzed by PCR-HRM with the reverse-specific primer strategy. In the studied time frame, the mycelium observed growing from the wood pieces did not have morphological characteristics allowing species identification. *Cyt. sorbicola* was less frequently recovered in the Santina and Regina sweet cherry varieties, with average values of 18.5% and 18.3%, respectively, on day seven (Table 3). In contrast, *Cyt. sorbicola* recovery from Lapins and Bing wood roughly doubled over the same period: 33.3% and 36.7% on average, respectively (Table 3). The recovery of *Cal. pulchella* was higher than *Cyt. sorbicola* in all sweet cherry varieties evaluated, with average percentages over 50% from day four of analysis (Table 3). In the Santina variety, the recovery of *Cal. pulchella* was lower on day seven, with 63.2% on average, followed by Regina, Bing, and Lapins, with 72.5%, 79.1%, and 89.7%, respectively (Table 3). Remarkably, the growth of *Cyt. sorbicola* and *Cal. pulchella* was slowed by 20% when comparing growth on days four to seven on wood of the Santina variety (Table 3). None of the pathogens were identified from the control wood, confirming the plant health of the material used in the experiments.

## 4. Discussion

The sweet cherry canker fungi *Cyt. sorbicola* and *Cal. pulchella* are a threat to the industry worldwide. Signs of *Cyt. sorbicola* and *Cal. pulchella* are widespread in sweet cherry orchards in the Chilean Central Valley, indicating that the implemented control measures are ineffective. Our work showed that *Cyt. sorbicola* and *Cal. pulchella* can be found in young or mature orchards and even coexist in the same cherry tree. This potential for coexistence adds another layer of challenge to managing these pathogens.

The genetics of sweet cherry tree varieties determine the fruit’s organoleptic characteristics and relevant agronomic traits [35,36]. In addition, the genetic background of each variety determines differential responses to abiotic and biotic stress [37,38,39]. The susceptibility of sweet cherry and other *Prunus* spp. has been evaluated for a limited group of economically relevant bacteria and fungi, including *Pseudomonas syringae* pv. *syringae* (*Pss*), *Erwinia amylovora*, and *Diaporthe amygdali*; however, these works show that the susceptibility depends on the species and the variety/genotype tested [12,40,41]. Coherently, our research showed that different sweet cherry tree varieties have variable susceptibility to *Cyt. sorbicola* and *Cal. pulchella,* and the susceptibility extent determined by wood necrosis is specific to each pathogen. It is noteworthy that the Lapins variety, which was less susceptible to wood decay fungi in our study, is highly vulnerable to the bacteria *E. amylovora*. Conversely, the Regina variety, the most prone to infection by *Cyt. sorbicola* and *Cal. pulchella*, is actually the most resistant to the *Pss* bacteria. Bacterial and fungal perception occurs through plant differential systems [42,43,44]. Our results are consistent with differential mechanisms conditioning susceptibility to pathogenic fungi and bacteria in cherry trees. Alternatively, signaling pathways relevant to bacteria and fungi may have segregated independently during the selection of sweet cherry tree varieties, favoring even the functional selection of more aggressive or pathogenic isolates. Although this hypothesis needs to be validated, it is an exciting avenue of research to be undertaken by breeders of sweet cherry varieties. This topic is crucial for cherry production in Chile, where *Pss* is as relevant as, or even more than, wood decay fungi from a phytosanitary perspective [45,46]. Furthermore, the climate crisis has caused changes in the optimal agroclimatic conditions for crops, leading to increased biotic stress in plants. A recent study demonstrated that cherry trees adjust their response to heat stress, negatively impacting their defense response to *Pss* [47]. Therefore, future works should thoroughly review the sweet cherry tree response to fungal and bacterial pathogens under stress conditions to align with the new field reality. 

Morphological analysis is the most commonly used strategy to identify *Cytospora* spp. and *Calosphaeria* spp. The time needed to develop key morphological characteristics by wood microorganisms in vitro leads to diagnoses that fall outside the time required to construct effective management programs. Molecular biology methods have been developed for *Cytospora* spp., including PCR-pathogen-specific, ddPCR, and LAMP methods, but not for *Calosphaeria* spp. [17,18,19]. Our research designed and developed a PCR-HRM assay for simultaneous detection of *Cyt. sorbicola* and *Cal. pulchella*. The three genotypes tested (*Cyt. sorbicola*, *Cal. pulchella*, and *Cyt. sorbicola* + *Cal. Pulchella*) provided melting profiles with distinct and consistent shapes, enabling easy discrimination between them. Nucleotide content, particularly the GC/AT ratio, determines the PCR-HRM design’s success [20,48,49]. Our strategies using universal primers or specific reverse primers were successful. Indeed, the specific reverse primer strategy generated melting profiles with greater differences, correlating with larger GC/AT ratio variations between the designed *Cyt. sorbicola* and *Cal. pulchella* fragments. Diagnosis depends on obtaining mycelium from four-day-old wood pieces, allowing fungi identification independent of the plant species. For example, *Cal. pulchella* has recently been identified as a cause of wood necrosis in peach trees in Chile [30]. Contaminants (other fungi) do not cause failures in the diagnostic system. Together, these tests can be completed in four days, significantly less than the morphology-based analyses that require between two and four weeks. Finally, this PCR-HRM diagnostic system can be used as a scaffold to include other microorganisms. Other species closely related to *Cytospora* that have been reported as problems for other *Prunus* spp. can be easily identified due to the conservation in the *ITS* region.

The PCR-HRM technique allowed us to evaluate the frequency of each pathogen in infected sweet cherry plants. Notably, recovery of *Cyt. sorbicola* and *Cal. pulchella* from wood samples showed no positive correlation between sweet cherry tree susceptibility and pathogen growth. However, the results revealed that *Cal. pulchella* has inherent advantages over *Cyt. sorbicola* in colonizing wood, suggesting a higher permanence in pruning remains. 

Our study reveals the differential susceptibility of sweet cherry tree varieties to *Cyt. sorbicola* and *Cal. pulchella*. The differential responses recorded depend on the plant genotype; therefore, specific control programs for each pathogen are required. Future studies should address the coexistence of *Cyt. sorbicola* and *Cal. pulchella*, including the plant defense response and changes in the aggressiveness of the fungi as a result of sharing the host. The PCR-HRM tool developed and validated in this study will enable decisions based on the pathogen’s presence in the field. In addition, the tool allows the orchard’s health status or nursery material to be checked.

## Figures and Tables

**Table 1:** Sequences used in the phylogenetic analysis of *Cyt. sorbicola* from sweet cherry trees.

Species	Strain	Host	Origen	GenBank Accession Number		
				*ITS*	*ACT*	*TEF-1α*
*Cytospora sorbicola*	KARE59	*Prunus dulcis*	California, USA	MG971861	MG972010	MG971572
*Cyt. sorbi*	MFLUCC 16-0631T	*Sorbus aucuparia*	Rusia	KY417752	KY417718	NA
*Cyt. sorbicola*	KARE621	*Prunus avium*	California, USA	MG971811	MG971961	MG971526
*Cyt. sorbicola*	KARE877	*Prunus avium*	California, USA	MG971825	MG971975	MG971540
*Cyt. sorbicola*	IRAN 4202C	*Malus domestica*	Iran	MW295654	MZ014514	MW394148
*Cyt. donetzica*	MFLUCC 16-0574T	*Rosa* sp.	Rusia	KY417731	KY417696	NA
*Cyt. leucostoma*	CFCC 50023	*Cornus alba*	China	KR045635	KU711003	KU710926
*Cyt. plurivora*	CBS 144239T	*Olea europaea*	California, USA	MG971861	MG972010	NA MG971572
*Cyt. parapersoonii*	T28.1T	*Prunus persica*	USA	AF191181	NA	NA
*Cyt. amygdali*	CBS 144233T	*Prunus dulcis*	California, USA	MG971853	MG972002	MG971659
*Cyt. olivacea*	CFCC 53176T	*Sorbus tianschanica*	China	MK673068	MK673038	MK672955
*Cyt. erumpens*	MFLUCC 16-0580T	*Salix _ fragilis*	Rusia	KY417733	KY417699	NA
*Cyt. mali-spectabilis*	CFCC 53181T	*Malus spectabilis*	China	MK673066	MK673036	MK672953
*Cyt. nivea*	CFCC 89642T	*Salix psammophila*	China	KF765684	KF765732	NA
*Cyt. translucens*	CXY 1351	*Populus davidiana*	China	KM034874	NA	NA
*Cyt. gigaspora*	CFCC 89634T	*Salix psammophila*	China	KF765671	KU711000	KU710923
*Cyt. paratranslucens*	MFLUCC 15-0506T	*Populus alba var. bolleana*	Rusia	KY417741	KY417707	NA
*Cyt. leucosperma*	CFCC 89622	*Pyrus bretschneideri*	China	KR045616	KU710988	KU710911
*Cyt. mali*	CFCC 50028	*Malus pumila*	China	MH933641	MH933548	MH933513
*Diaporthe vaccinii*	CBS 160.32	*Vaccinium macrocarpon*	USA	KC343228	JQ807297	KC343954

**Table 2:** Primer sequences used in this study.

Locus	Primer Name	Primer Sequence (5′→3′)	Reference
*Internal Transcribed Spacer*	*ITS1F*	TCCGTAGGTGAACCTGCGG	[24]
	*ITS4*	TCCTCCGCTTATTGATATGC	[24]
*Actin*	*ACT512F*	ATGTGCAAGGCCGGTTTCGC	[25]
	*ACT-783R*	TACGAGTCCTTCTGGCCCAT	[25]
*Translation elongation factor 1-alpha*	*EF1-728*	CATCGAGAAGTTCGAGAAGG	[25]
	*EF1-986*	TACTTGAAGGAACCCTTACC	[25]
*Internal Transcribed Spacer*	*Calo-Cytos_For*(*ITS*)	GGGTTGAAATGACGCTCG	This study
	*Calos-Cytos_Rev*(*ITS*)	CTGGCATCGATGAAGAACG	This study
	*Calo-Cytos_For-univ1*(*ITS*)	CATTTCGCTRCGTTCTTCATC	This study
	*Cyto_Rev-esp*(*ITS*)	GGCCCACTAAACTCTTGTATT	This study
	*Calos _Rev-esp*(*ITS*)	GTCCTCTGAGCCAACTGAAA	This study

**Table 3:** *Cyt. sorbicola* and *Cal. pulchella* recovery from inoculated sweet cherry wood.

	*Cyt. sorbicola*			*Cal. pulchella*		
	4 Days (%)	7 Days (%)	Δ ^1^ (%)	4 Days (%)	7 Days (%)	Δ ^1^ (%)
Santina	14.8	18.5	20.0	50.9	63.2	19.5
Lapins	33.3	33.3	0.0	87.9	89.7	2.0
Bing	35.0	36.7	4.6	79.1	79.1	0.0
Regina	18.3	18.3	0.0	70.0	72.5	3.4

^1^ Variation between days 7 and 4.

## Data Availability

Data are the property of the Laboratory of Fruit and Molecular Phytopathology of the Faculty of Agronomic Sciences, University of Chile, Santiago, Chile. Any requests should be directed to Marcela Esterio (Principal Investigator).
